# 2,2',2''-Terpyridine-Catalyzed Synthesis of Cyclic Carbonates from Epoxides and Carbon Dioxide under Solvent-Free Conditions

**DOI:** 10.3390/ijms15069945

**Published:** 2014-06-04

**Authors:** Huixin Liu, Renqin Zeng, Ruimao Hua

**Affiliations:** Department of Chemistry, Tsinghua University, Beijing 100084, China; E-Mails: liuhuixin9015@163.com (H.L.); zengrq@mails.tsinghua.edu.cn (R.Z.)

**Keywords:** carbon dioxide, epoxide, cyclic carbonates, terpyridine

## Abstract

An efficient coupling reaction of epoxides with CO_2_ affording cyclic carbonates with the use of 2,2',2''-terpyridine as catalyst under solvent-free conditions has been developed.

## 1. Introduction

**Scheme 1:**
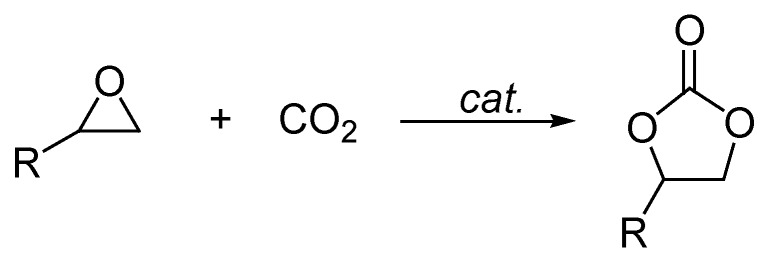
Synthesis of cyclic carbonates from epoxides and CO_2_.

The coupling reaction of epoxides with CO_2_ is an atom-economic and well-known process for the synthesis of five-membered cyclic carbonates (Scheme 1), which have been widely applied as the aprotic polar solvents [1], electrolytes for lithium ion batteries [2], precursors for organic synthesis [3], and polymers [4]. Therefore, a variety of catalytic systems have been reported to catalyze this transformation including alkali metal compounds [5,6,7], ionic liquids [8,9,10], transition metal complexes [11,12,13,14,15], and heterogeneous catalysts [16,17,18,19].

On the other hand, recently, the catalytic reactions based on the use of small organic molecules as catalysts have been greatly developed with the significant advantages of metal-free procedure, lack of sensitivity to moisture and oxygen, affordability, low cost and low toxicity [20,21]. Recently, the reaction between amine and CO_2_ is considered to be one of the methods for the storage of CO_2_ [22], and it has been also disclosed that the quaternary ammonium salts [23] and amines [24] are the efficient catalysts for the reaction of epoxides with CO_2_ to give cyclic carbonates. In addition, our previous work revealed that *N*,*N*-dimethylformamide (DMF) could catalyze the same reaction efficiently [25]. In continuation of our interest in developing efficient catalytic systems using simple and cheap organic compounds as catalysts in the coupling reaction of epoxides with CO_2_, we therefore have investigated the catalytic activity of nitrogen-containing organic compounds such as amines, anilines, amides and pyridines, and found that 2,2',2''-terpyridine is an excellent organocatalyst to provide an alternative metal-free catalytic system for the conversion of CO_2_ to cyclic carbonates.

## 2. Results and Discussion

Table 1 concludes the catalytic activity of a variety of nitrogen-containing organic compounds in the coupling reaction of 1-chloro-2,3-epoxypropane (**1a**) with CO_2_ (3.0 MPa of initial pressure) in an autoclave in the presence of 10 mol % of organocatalysts at 130 °C for 20 h under solvent-free conditions. In general, all the used nitrogen-containing compounds showed the catalytic activity to promote the coupling reaction to give the desirable product of 4-cholromethyl-[1,3]dioxolan-2-one (**2a**) in fair to good yields. As was observed for the formation of **2a**, trialkyl tertiary amines of Et_3_N and Bu_3_N showed modest catalytic activity to give **2a** in 48% and 53% yields, respectively (entries 1,2). Although TEMED (*N,N,N',N'*-tetramethylethylenediamine), DBU (1,8-diazabicyclo[5.4.0]-undec-7-ene) and DABCO (1,4-diazabicyclo[2.2.2]octane) contains two nitrogen atoms, they displayed the considerable different catalytic activities, and DABCO showed higher catalytic activity due possible to its higher nucleophilicity resulting from the alkyl groups not to disturb the lone pairs (entries 3,4 *vs.* entry 5). In addition, TBD (1,5,7-Triazabicyclo[4.4.0]dec-5-ene), which has three different nitrogen atoms showed moderate catalytic activity to give **2a** in 56% yield (entry 6). In the cases of anilines, the catalytic activity was enhanced by increasing the number of methyl groups to nitrogen atom (entries 7–9). However, unexpectedly, benzamide, *N,N*-dimethylbenzamide and 2,3,4,5,6-pentafluorobenzamide had similar catalytic activities to catalyze the coupling reaction to give **2a** in good yields (entries 10–12). When six-membered *N*-heterocyclic compounds such as pyridine, 2-methylpyridine and 2-(dimethylamino)pyridine were used, **2a** could be obtained in modest to good yields (entries 13–15). 2-Phenylpyrimidine with two nitrogen atoms catalyzed the reaction to afford **2a** in 79% yield (entry 16), and the best yield in product **2a** was obtained in the presence of 2,2',2''-terpyridine (entry 17). The reason that 2,2',2''-terpyridine had high catalytic activity might be resulted from its unique structure and synergy effects of three pyridyl groups bonded by position 2.

In addition, in order to optimize the catalytic system using 2,2',2''-terpyridine as catalyst, the effect of reaction temperature, catalyst dosage, reaction time and pressure of CO_2_ were also investigated. As shown in Table 2, the formation of **2a** was greatly affected by reaction temperature, and when the reaction was performed at 110 °C, the yield of **2a** was greatly decreased to 72% (entry 1). However, at 130 °C, **2a** could be obtained in similar yields by either using less amount of catalyst (5.0 mol % (entry 2) or 1.0 mol % (entry 3) *vs.* entry 16 of Table 1), or decreasing the reaction time from 20 to 10 h (entry 5), although further decreasing the catalyst dosage (0.5 mol % of catalyst, entry 4) and the reaction time (entry 6) or decreasing the pressure of CO_2_ resulted in the decrease of product yield (entry 7). In addition, increase of CO_2_ pressure could not improve the yield of **2a** (entry 8). Therefore, the reaction conditions indicated in entry 5 of Table 2 are selected as the optimized conditions for the reactions of a variety of epoxides with CO_2_.

**Scheme 2:**
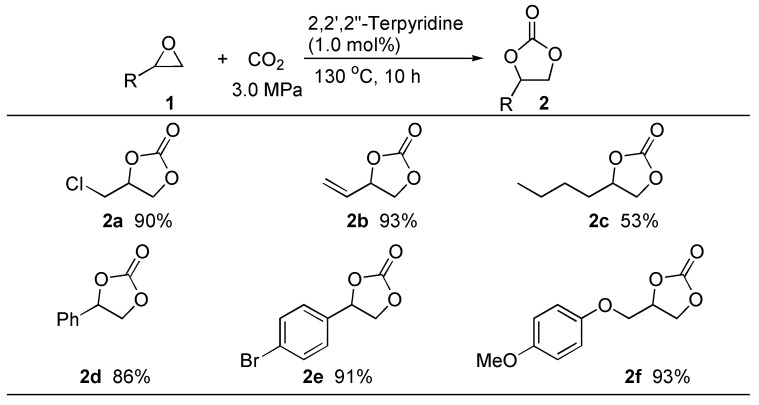
Coupling reaction of epoxides with CO_2_ in the presence of 2,2',2''-terpyridine ^a^. ^a^ Reactions were carried out using 5.0 mmol of **1** and the yield of **2** is isolated yields.

To examine the substrate scope of the present catalytic system, several representative epoxides were subjected to the optimized reaction conditions as shown in Scheme 2, and found that the monoalkyl- and monoaryl-substituted epoxides could undergo the coupling reaction with CO_2_ giving the corresponding cyclic carbonates in good to high yields. It is worth noting that vinyl-substituted epoxide underwent the reaction smoothly to give the vinyl cyclic carbonate, which is expected to be a useful monomer for synthesis of functional polymer. However, unfortunately, the internal epoxide such as 2,3-epoxybutane and 1,2-epoxycyclohexane showed very low reactivity due possible to their steric hindrance.

**Table 1:** Catalytic activity of nitrogen-containing compounds in the coupling of 1-chloro-2,3-epoxypropane (**1a**) with CO_2_ under solvent-free conditions ^a^.

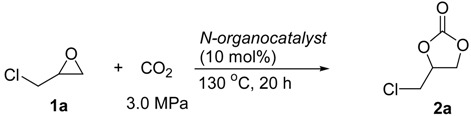					
Entry	Organocatalyst	Yield (%) ^b^	Entry	Organocatalyst	Yield (%) ^b^
1	Et_3_N	48	12	F_5_C_6_CONH_2_	71
2	Bu_3_N	53	13	pyridine	59
3	TEMED	35	14	2-methylpyridine	63
4	DBU	32	15	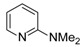	71
5	DABCO	46			
6	TBD	56	16	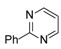	79
7	PhNH_2_	59			
8	PhNHMe	70			
9	PhNMe_2_	81	17	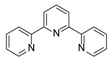	88
10	PhCONH_2_	70			
11	PhCONMe_2_	74			

^a^, The reactions were carried out using 5.0 mmol of **1a** and 10 mol % of catalyst in a 25-mL autoclave with CO_2_ at 130 °C for 20 h; ^b^, Yields of **2a** are based on GC by using *n*-C_18_H_38_ as internal standard.

**Scheme 3:**
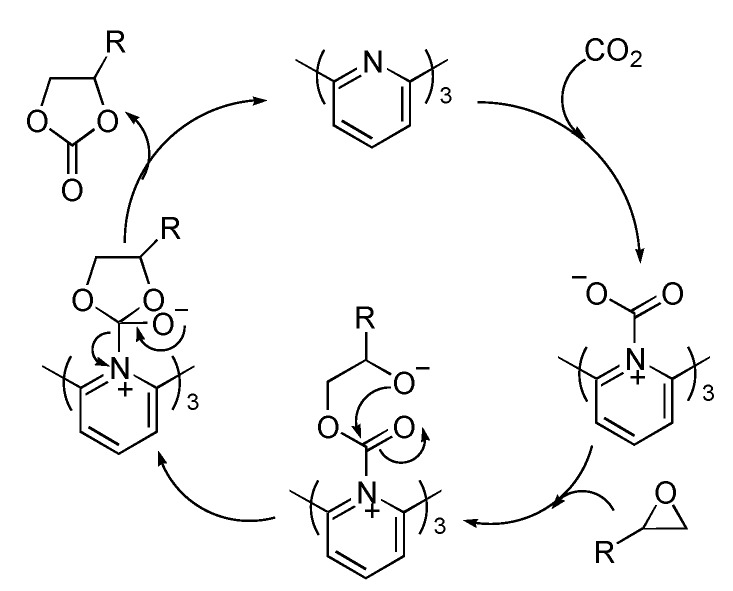
A proposed mechanism for the formation of cyclic carbonate.

The proposed mechanism for the coupling reaction of epoxides with CO_2_ to produce cyclic carbonates catalyzed by 2,2',2''-terpyridine is shown in Scheme 3. It involves the nucleophilic addition of nitrogen atom(s) of 2,2',2''-terpyridine to CO_2_, and the ring-opening reaction of epoxide with nucleophilic intermediate via C-O bond cleavage, followed by intramolecular nucleophilic addition to construct the five-membered ring, and finally, to give cyclic carbonate and regenerate catalyst. All the steps are the traditional and well-known transformation.

**Table 2:** Effect of reaction conditions on the formation of 4-cholromethyl-[1,3]dioxolan-2-one (**2a**) using 2,2',2''-terpyridine as catalyst ^a^.

Entry	Temp (°C)	Catalyst (mol %)	Time (h)	Pressure (MPa)	Yield (%) ^b^
1	110	10	20	3.0	72
2	130	5	20	3.0	88
3	130	1	20	3.0	87
4	130	0.5	20	3.0	68
5	130	1	10	3.0	90
6	130	1	6	3.0	80
7	130	1	10	2.5	81
8	130	1	10	4.0	89

^a^, The reactions were carried out using 5.0 mmol of **1a** in a 25-mL autoclave with CO_2_; ^b^, Yields of **2a** are based on GC by using *n*-C_18_H_38_ as internal standard.

## 3. Experimental Section

### 3.1. General Methods

All organic starting materials and organocatalysts are analytically pure and used without further purification. Nuclear magnetic resonance (NMR) spectra were recorded on a JEOL ECA-300 spectrometer (Tokyo, Japan) using CDCl_3_ as solvent at 298 K. ^1^H-NMR (300 MHz) chemical shifts (δ) were referenced to internal standard TMS (for ^1^H, δ = 0.00 ppm). ^13^C-NMR (75 MHz) chemical shifts were referenced to internal solvent CDCl_3_ (for ^13^C, δ = 77.16 ppm). Mass spectra (MS) were obtained on a Shimadzu GCMS-QP2010S (Shimadzu, Tokyo, Japan).

### 3.2. A Typical Experiment for the Synthesis of 4-Chloromethyl-[1,3]dioxolan-2-one (2a)

1-Chloro-2,3-epoxypropane (**1a**) (462.6 mg, 5.0 mmol) and 2,2',2''-terpyridine (0.05 mmol, 1.0 mol %) were charged in a 25 mL-autoclave, and then CO_2_ was introduced at an initial pressure of 3.0 MPa at room temperature, and the mixture was heated at 130 °C with stirring for 10 h. After the reaction, the autoclave was cooled to room temperature, CO_2_ was released slowly. To the obtained reaction mixture, 1.0 mmol of octadecane (as an internal standard material for GC analysis) and 4.0 mL of CH_2_Cl_2_ were added with stirring, and then the resulting mixture was analyzed by GC and GC-MS. **2a** was obtained in 90% yield (614.4 mg, 4.5 mmol) by Kugelrohr distillation. **2a**–**c** was isolated by Kugelrohr distillation, and **2d**–**f** were purified by flash column chromatography on silica gel with petroleum ether as eluent.

All the products (**2a**–**f**) were known compounds and identified by their ^1^H-NMR, ^13^C-NMR and GC-MS. As exampled, the characterization data of **2a** is reported as follow: ^1^H-NMR (300 MHz, CDCl_3_) δ 4.95–5.02 (m, 1H), 4.58 (dd, 1H, *J* = 8.7, 8.2 Hz), 4.39 (dd, 1H, *J* = 8.7, 5.8 Hz), 3.79–3.73 (m, 2H); ^13^C-NMR (75 MHz, CDCl_3_) δ 154.4, 74.4, 67.1, 44.0; GCMS *m*/*z* (% rel. intensity) 136 (M^+^, 0.5), 87 (100), 62 (14), 57 (9), 49 (13).

## 4. Conclusions

In summary, 2,2',2''-terpyridine was proven to be an efficient organocatalyst for the coupling reaction of epoxides with CO_2_ to afford cyclic carbonates under solvent-free and metal-free conditions. The present process represents a simple and green catalytic system for the activation and conversion of CO_2_ into valuable organic compounds.
